# Long‐Term Survival of Two Versus Three Courses of Preoperative Cisplatin and Fluorouracil Plus Docetaxel for Locally Advanced Esophageal Cancer: A Multicenter Randomized Phase II Trial

**DOI:** 10.1002/ags3.70036

**Published:** 2025-05-10

**Authors:** Takahito Sugase, Hiroshi Miyata, Takashi Kanemura, Norihiro Matsuura, Tomoki Makino, Makoto Yamasaki, Koji Tanaka, Kotaro Yamashita, Kota Momose, Osamu Shiraishi, Keijiro Sugimura, Masaaki Motoori, Kazumasa Fujitani, Atsushi Takeno, Motohiro Hirao, Yutaka Kimura, Taroh Satoh, Masahiko Yano, Yuichiro Doki, Takushi Yasuda

**Affiliations:** ^1^ Department of Gastroenterological Surgery Osaka International Cancer Institute Osaka Japan; ^2^ Department of Gastroenterological Surgery Osaka University Graduate School of Medicine Osaka Japan; ^3^ Department of Surgery Kansai Medical University Hirakata Japan; ^4^ Department of Surgery Kindai University Faculty of Medicine Osaka Japan; ^5^ Department of Surgery Kansai Rosai Hospital Amagasaki Japan; ^6^ Department of Surgery Osaka General Medical Center Osaka Japan; ^7^ Department of Surgery, NHO Osaka National Hospital Osaka National Hospital Osaka Japan; ^8^ Department of Surgery Kindai University Nara Hospital Nara Japan; ^9^ Department of Frontier Science for Cancer and Chemotherapy Osaka University Graduate School of Medicine Osaka Japan; ^10^ Department of Surgery Suita Municipal Hospital Osaka Japan

**Keywords:** DCF, esophageal cancer, long‐term survival, preoperative chemotherapy, three courses, two courses

## Abstract

**Background:**

Preoperative chemotherapy with cisplatin, fluorouracil, and docetaxel (DCF) is one of the neoadjuvant treatments for locally advanced esophageal squamous cell carcinoma (ESCC). However, the optimal number of DCF cycles remains unknown. This multi‐institutional, randomized, phase II trial aimed to investigate the long‐term survival outcomes of two versus three courses of DCF.

**Methods:**

A total of 180 patients with locally advanced ESCC from six institutions were randomly assigned to receive either two (*N* = 91) or three (*N* = 89) courses of DCF administered every 3 weeks prior to surgery. Long‐term survival outcomes were compared between the two regimens.

**Results:**

Baseline characteristics were well balanced between the two groups. The 5‐year overall survival (OS) and progression‐free survival (PFS) rates for the three and two course groups were 70.7% vs. 63.8% (hazard ratio (HR) = 0.91, *p* = 0.717) and 63.3% vs. 60.0% (HR = 0.94, *p* = 0.810) respectively, with no significant differences observed. The per‐protocol analysis exhibited similar results, with OS rates of 71.1% vs. 68.8% (HR = 0.90, *p* = 0.702) and PFS rates of 63.6% vs. 65.4% (HR = 0.92, *p* = 0.773). Recurrence patterns were also similar between the groups. Subgroup analysis revealed that non‐responders in the three course DCF group had significantly worse long‐term survival outcomes, whereas the two course DCF group exhibited minimal trends in this regard. Conversely, patients aged < 65 years or those with favorable clinical responses in the three course group demonstrated improved long‐term survival outcomes.

**Conclusion:**

Two courses of preoperative DCF followed by radical esophagectomy can be one of the potential treatment strategies for locally advanced ESCC.

**Trial Registration:**

ClinicalTrials.gov identifier: UMIN 000015788.

## Graphical abstract

This multi‐institutional, randomized, phase II trial aimed to investigate the long‐term survival outcomes of two versus three courses of DCF for locally advanced esophageal squamous cell carcinoma (ESCC). The 5‐year overall survival and progression‐free survival rates for the three and two course groups were 70.7% vs. 63.8% (hazard ratio (HR) = 0.91, *p* = 0.717) and 63.3% vs. 60.0% (HR = 0.94, *p* = 0.810) respectively, with no significant differences observed.

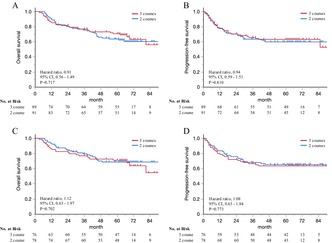

## 1 Introduction

Esophageal cancer ranks tenth among the most commonly diagnosed cancers and sixth as a leading cause of cancer‐related mortality worldwide [1]. Despite advancements in multidisciplinary treatments, including surgery, radiotherapy, chemotherapy, and immunotherapy [2, 3, 4, 5, 6, 7, 8, 9], the prognosis of patients with esophageal cancer remains poor. Preoperative treatment followed by surgery has been employed to manage advanced esophageal cancer, aiming to achieve a higher R0 resection rate, eliminate micrometastases, and reduce the risk of recurrence [2, 3, 5, 10].

Since the CROSS trial, neoadjuvant chemoradiotherapy has become the standard treatment for patients with resectable esophageal cancer in Western countries [3, 4]. The JCOG9907 trial demonstrated that preoperative chemotherapy with two courses of cisplatin and 5‐fluorouracil (CF) improved long‐term survival compared with postoperative CF in patients with locally advanced esophageal squamous cell carcinoma (ESCC). Consequently, neoadjuvant chemotherapy (NAC) has emerged as an alternative treatment [10]. To improve treatment outcomes, including addressing microscopic metastatic disease, achieving tumor downstaging, and increasing resectability, triplet NAC regimens and CF plus docetaxel (DCF) have been introduced. Our multicenter randomized phase II trial (OGSG1003) revealed that two courses of NAC with DCF prolonged the recurrence‐free survival (RFS) and overall survival (OS) in patients with resectable advanced ESCC [11, 12]. Recently, a randomized phase III trial (JCOG1109) focused on preoperative treatment for resectable ESCC and compared preoperative DCF with CF (standard arm) and CF plus radiotherapy (RT). This study confirmed that three courses of DCF followed by esophagectomy resulted in statistically significant improvements on OS compared to that of the two courses of CF. Conversely, neoadjuvant treatment with two courses of CF plus RT did not demonstrate a significant survival advantage over two courses of CF [5]. Based on the findings of the JCOG1109 trial, the Japan Esophageal Society has strongly recommended three courses of DCF as the new standard treatment for locally advanced ESCC.

In daily clinical practice, protocols involving two cycles of cisplatin‐based NAC have been frequently used in several clinical trials [2, 3, 4, 11, 12]. By contrast, the JCOG1109 trial compared three courses of DCF, two courses of CF (standard arm), and two courses of CF plus RT [5]. However, the optimal number of NAC cycles for ESCC has not yet been established, and the effect of an additional course of NAC on the response rate or survival compared with that of the two standard courses remains unclear.

Hence, this multi‐institutional, randomized phase II trial aimed to compare two and three courses of NAC with a DCF regimen to determine the optimal number of NAC cycles required for treating resectable advanced ESCC. Our previous studies indicated that two and three courses of DCF regimens in the NAC setting were similarly feasible. Additional courses of DCF improved NAC responses without increasing the incidence of adverse events or postoperative morbidity. However, the short‐term survival rates were comparable between the two groups [13, 14]. The present study compared the long‐term survival outcomes between the two arms of this randomized phase II trial.

## 2 Methods

### 2.1 Patients

The eligibility criteria and results of pretreatment evaluations have been described in detail in previous studies [13, 14]. Patients aged ≥ 20 years, with a performance status score of 0–1, who were histologically diagnosed with ESCC, and who exhibited adequate primary organ function were considered eligible for the study. The ESCC stages included cT1‐4a N0‐3 M0 and/or M1LYM metastases confined to the supraclavicular lymph nodes, based on the seventh edition of the Union for International Cancer Control TMN classification [15]. Patients who achieved a complete response (CR) or partial response (PR) were classified as responders, whereas those with stable disease (SD) or progressive disease (PD) were classified as non‐responders [14]. All patients provided written informed consent to participate in the study. The study was approved by the institutional review boards of the six participating hospitals prior to patient enrollment. The study was conducted in accordance with the principles of the Helsinki Declaration and registered with the University Hospital Medical Information Network Clinical Trials Registry (identification number: UMIN 000015788).

### 2.2 Study Treatment

In this open‐label randomized phase II trial, all eligible patients were randomly assigned to receive either two or three courses of DCF. Each DCF course included docetaxel 70 mg/m^2^ (1 h intravenous infusion) plus cisplatin 70 mg/m^2^ (1 h intravenous infusion) on day 1, followed by 5‐fluorouracil 700 mg/m^2^ (continuous intravenous infusion) for 5 days. The courses were administered every 3 weeks [16, 17, 18, 19, 20]. Randomization was stratified according to institution, cT stage, and cN stage using the least‐squares method.

To manage adverse effects, the doses of the likely causal agents were adjusted in subsequent cycles as follows [12, 13]: for grade 4 leukopenia or neutropenia, febrile neutropenia, or grade 3 thrombocytopenia, the doses of all chemotherapy agents were reduced by 20%; for grade 3 stomatitis or diarrhea, the 5‐FU and docetaxel doses were reduced by 20%; and for grade 2 nephrotoxicity, the cisplatin dose was reduced by 20%. A second cycle was administered unless disease progression or unacceptable toxicity occurred.

Surgery was scheduled 3–6 weeks after the last chemotherapy cycle. Patients underwent subtotal esophagectomy with either two‐ or three‐field lymphadenectomy with curative intent, performed via the right thoracotomy or thoracoscopic approach [16, 18, 20, 21, 22]. Transhiatal esophagectomy was not permitted. Regional lymphadenectomy included the mediastinal, perigastric, and celiac nodes, whereas distant lymphadenectomy included the cervical nodes.

### 2.3 Evaluation of Clinical Responses

The clinical evaluation of tumor response after chemotherapy was performed using contrast‐enhanced 64‐slice computed tomography (CT) or esophagoscopy when tumors were undetectable or unmeasurable by CT. The primary tumor size was assessed bi‐dimensionally by measuring the greatest diameter and its perpendicular length. Lymph node response was evaluated by measuring the minor axis of metastatic lymph nodes with a diameter of ≥ 10 mm, and the total sum was calculated according to the Response Evaluation Criteria in Solid Tumors (RECIST) guidelines. The chemotherapeutic response was determined based on the percentage reduction in the primary tumor area and the sum of the minor axes of metastatic lymph nodes [13]. Clinical responses were classified as follows: CR, defined as complete regression of the disease confirmed by CT and endoscopy; PR, defined as a reduction of more than 50% in the primary tumor size and more than 30% in the size of metastatic lymph nodes; PD, defined as an increase of more than 25% in the primary tumor size or the appearance of new lesions, or an increase of more than 20% in metastatic lymph nodes; and SD, defined as cases not meeting the criteria for PR or PD. The overall clinical response was determined based on the worst outcome observed in either the primary tumor or the lymph nodes.

### 2.4 Follow‐Up

Patients were followed up every 3 months for the first 2 years after random assignment, every 6 months for the next 3 years, and annually [22, 23, 24]. Disease recurrence was categorized as locoregional (in the esophageal bed, anastomotic site, or regional lymph nodes) or distant (in non‐regional lymph nodes, excluding supraclavicular lymph nodes, or distant organs) [18, 25]. The histopathological tumor response was evaluated based on the Japanese Society for Esophageal Disease criteria. Briefly, the evaluations were classified into five categories according to the proportion of tumors affected by degeneration or necrosis [20, 26].

### 2.5 Statistical Analysis

The sample size for this study was calculated as follows: 164 patients were required to detect an increase in the 2‐year PFS, from 55% in the two course group to 70% in the three course group, with 80% power to identify a significant difference between the groups and a 10% type I error. Assuming an approximate dropout rate of 10%, 180 patients were enrolled. Continuous variables were expressed as medians and ranges. Nonparametric variables were compared between the groups using the Mann–Whitney *U* test. Categorical data were expressed as frequencies (percentages) and compared between the groups using Fisher's exact test or Pearson's *χ* ^2^ test. The level of significance was set at a *p* value of 0.05. Progression‐free survival (PFS) and OS were calculated from the date of random assignment, estimated using the Kaplan–Meier method, and compared using the log‐rank test on an intent‐to‐treat basis. To assess the effects of the contributing factors, we calculated the hazard ratios (HR) and 95% confidence intervals (CI).

## 3 Results

### 3.1 Patient Characteristics and Chemotherapy Outcomes

**FIGURE 1:**
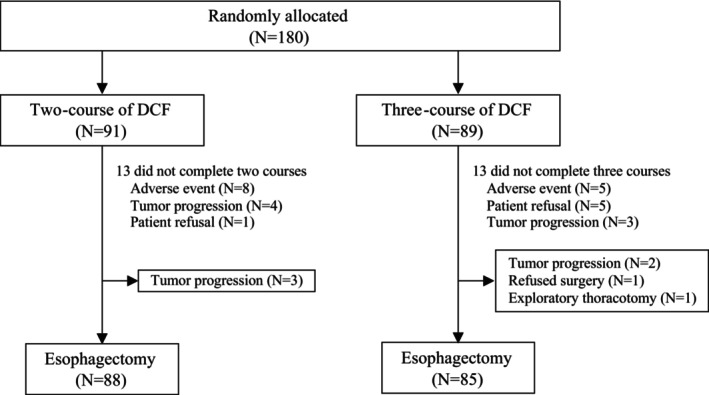
The Consolidated Standards of Reporting Trials diagram presents the patient allocation, treatment, and outcomes. DCF, Docetaxel, cisplatin, and 5‐fluorouracil.

The clinicopathological characteristics, treatment outcomes, and histopathological findings have been reported in previous studies [13, 14]. A total of 180 patients from six institutions were randomly assigned to receive either two (*N* = 91) or three (*N* = 89) courses of DCF between July 2014 and December 2018. The number of patients enrolled in each hospital ranged from 4 to 73, with a mean of 30. In the two course DCF group, 78 (86%) patients completed both courses of preoperative DCF. The reasons for not completing both chemotherapy courses included severe adverse effects (*N* = 8), PD (*N* = 4), and patient refusal (*N* = 1). In the three course DCF group, 76 patients (85%) completed all three courses. The reasons for the non‐completion of planned NAC courses in this group were severe adverse effects (*N* = 5), patient refusal (*N* = 5), and PD (*N* = 3) (Figure 1).

The patients were predominantly men (85%), with a median age of 67 years (range, 37–79 years). Most patients developed tumors in the middle or lower thorax (84%), with the majority classified as having cStage III disease (53%). The treatment groups were well‐matched in terms of baseline characteristics, including age, sex, performance status, body mass index, tumor markers, tumor location, and cStage. The groups exhibited comparable overall rates of grade ≥ 4 adverse events (75% vs. 80%, *p* = 0.419). At the end of the scheduled chemotherapy, the three course DCF group showed significantly higher rates of overall clinical response compared with the two course DCF group (43% vs. 65%, *p* = 0.003). Although the three course DCF group showed a relatively higher pathological CR rate than the two course DCF group (6% vs. 12%), the two groups showed similar histological responses for the primary tumor (*p* = 0.898) (Table 1).

**TABLE 1:** Patient characteristics, treatment outcomes, and histopathological findings.

	2‐course DCF		3‐course DCF		*p*
	*N* = 91		*N* = 89		
*Patients characteristics*					
Age, years, median (range)	67	(37–79)	67	(44–79)	1.000
Sex, *n* (%)					0.786
Male	78	(86)	75	(84)	
Female	13	(14)	14	(16)	
Performance status (ECOG), *n* (%)					0.836
0	82	(90)	81	(91)	
1	9	(10)	8	(9)	
BMI, kg/m^2^, median (range)	20.9	(16.0–30.1)	21.6	(14.2–30.9)	0.349
Tumor marker (SCC 1.6≦), *n* (%)	31	(34)	32	(36)	0.791
Tumor location, *n* (%)					0.587
Upper	16	(18)	13	(15)	
Middle/Lower	75	(82)	76	(85)	
cT stage, *n* (%)					0.920
1–2	28	(31)	28	(31)	
3–4	63	(69)	61	(69)	
cN stage, *n* (%)					0.908
0–1	73	(80)	72	(81)	
2–3	18	(20)	17	(19)	
cStage, *n* (%)					0.465
I—II	32	(35)	36	(41)	
III—IV	59	(65)	44	(59)	
*Treatment outcomes*					
Completion of planned courses, *n* (%)	78	(86)	76	(85)	0.951
Dose reduction during planned courses, *n* (%)	63	(69)	70	(79)	0.150
Overall adverse events, *n* (%)					
≧ grade 4	68	(75)	71	(80)	0.419
Clinical treatment response, *n* (%)					
CR	0	(0)	1	(1)	
PR	39	(43)	57	(64)	
SD	49	(54)	29	(33)	
PD	3	(3)	2	(2)	
RR	39	(43)	58	(65)	0.003
*Histopathological findings*					
ypStage, *n* (%)					0.077
0	5	(6)	11	(12)	
I	19	(21)	12	(14)	
II	33	(36)	24	(27)	
III	28	(31)	29	(33)	
IV	3	(3)	9	(10)	
Not resected	3	(3)	4	(4)	
Histopathological response, *n* (%)					0.898
Grade 0	3	(3)	2	(2)	
Grade 1a	27	(30)	31	(35)	
Grade 1b	23	(25)	18	(20)	
Grade 2	27	(30)	21	(24)	
Grade 3	8	(9)	13	(15)	
Not resected	3	(3)	4	(4)	

Abbreviations: CR, complete response; ECOG, eastern cooperative oncology group; PD, progressive disease; PR, partial response; RR, response rate (CR + PR); SCC, squamous cell carcinoma; SD, stable disease.

### 3.2 Long‐Term Survival

**FIGURE 2:**
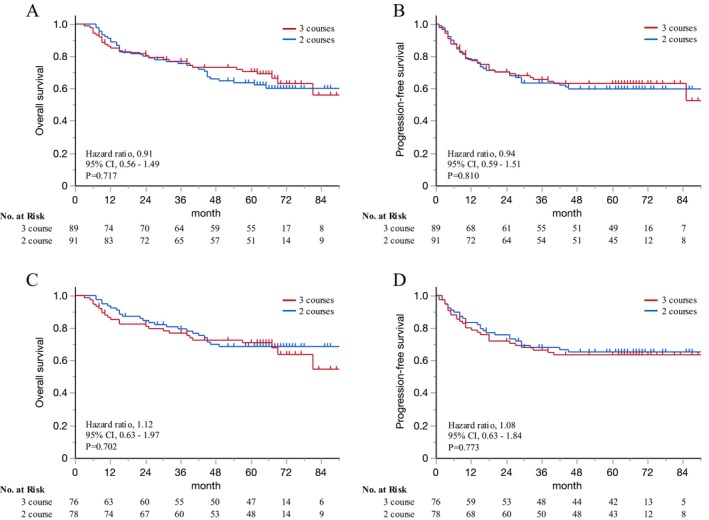
Survival analysis: Intention‐to‐treat analysis of the (A) overall survival and (B) progression‐free survival, and per‐protocol analysis of the (C) overall survival and (D) progression‐free survival.

The intention‐to‐treat (ITT) analysis showed 5‐year OS rates of 70.7% in the three course DCF group and 63.8% in the two course DCF group (HR: 0.91, 95% CI: 0.56–1.49, *p* = 0.717; Figure 2A). The 5‐year PFS rates were similar between the two groups: 63.3% in the three course DCF group and 60.0% in the two course DCF group (HR: 0.94, 95% CI: 0.59–1.51, *p* = 0.810; Figure 2B). The per‐protocol analysis revealed similar 5‐year OS rates between the three course (*n* = 76) and two course (*n* = 78) DCF groups (71.1% and 68.8%, respectively; HR: 0.90, 95% CI: 0.51–1.58, *p* = 0.702; Figure 2C), as well as comparable 5‐year PFS rates (63.6% and 65.4%, respectively; HR: 0.92, 95% CI: 0.54–1.58, *p* = 0.773; Figure 2D).

### 3.3 Recurrences

The two groups showed similar overall disease recurrence rates (31% vs. 30%, *p* = 0.494). No significant differences were observed in the locoregional or distant recurrence rates between the two groups (locoregional: 10% vs. 8%, *p* = 0.426; distant: 24% vs. 25%, *p* = 0.518). Additionally, the proportion of patients who developed distant metastases was similar between the two groups (Table 2).

**TABLE 2:** Patterns of postoperative recurrences in patients who underwent R0/1 resection.

	All	2‐course DCF		3‐course DCF		*p*
		*N* = 91		*N* = 89		
Overall	52	27	(31%)	25	(30%)	0.494
Locoregional recurrence	16	9	(10%)	7	(8%)	0.426
Distant recurrence	42	21	(24%)	21	(25%)	0.518
Lymph node	18	10	(11%)	8	(10%)	0.433
Lung	11	6	(7%)	5	(6%)	0.525
Bone	8	5	(6%)	3	(4%)	0.380
Liver	6	4	(5%)	2	(2%)	0.358
Brain	3	0	(0%)	3	(4%)	0.116
Pleura	3	2	(2%)	1	(1%)	0.513
Others	4	1	(1%)	3	(4%)	0.297

### 3.4 Subgroup Analysis

**FIGURE 3:**
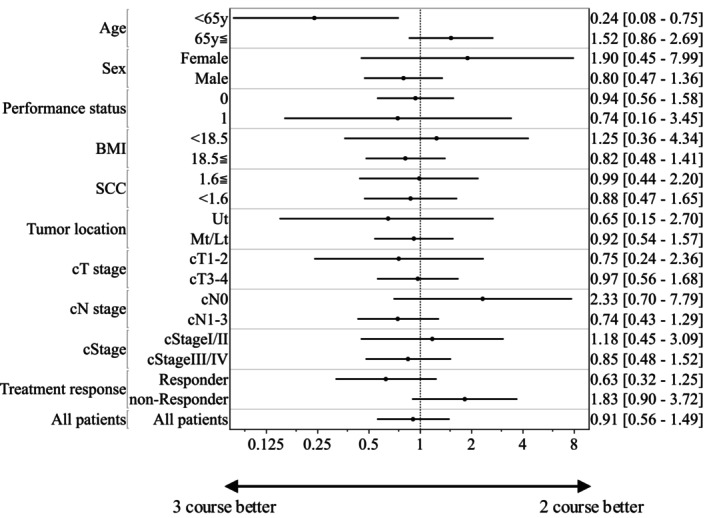
The forest plot illustrates hazard ratios (dots) and 95% confidence intervals (lines) for factors that may influence the overall survival. The analysis included 180 patients with esophageal cancer, and the factors represent baseline characteristics.

Figure 3 presents the results of the subgroup analysis of the baseline characteristics and clinical treatment responses of 180 patients with ESCC. No significant difference was found between the two and three course DCF regimens across subgroups defined by sex, performance status, body mass index, tumor markers, tumor location, cT stage, cN stage, cStage, or treatment response. However, in the subgroup of patients aged ≤ 65 years, the three course DCF regimen provided a survival benefit over the two course regimen (HR: 0.24, 95% CI: 0.08–0.75, *p* = 0.014; Figure 3).

**FIGURE 4:**
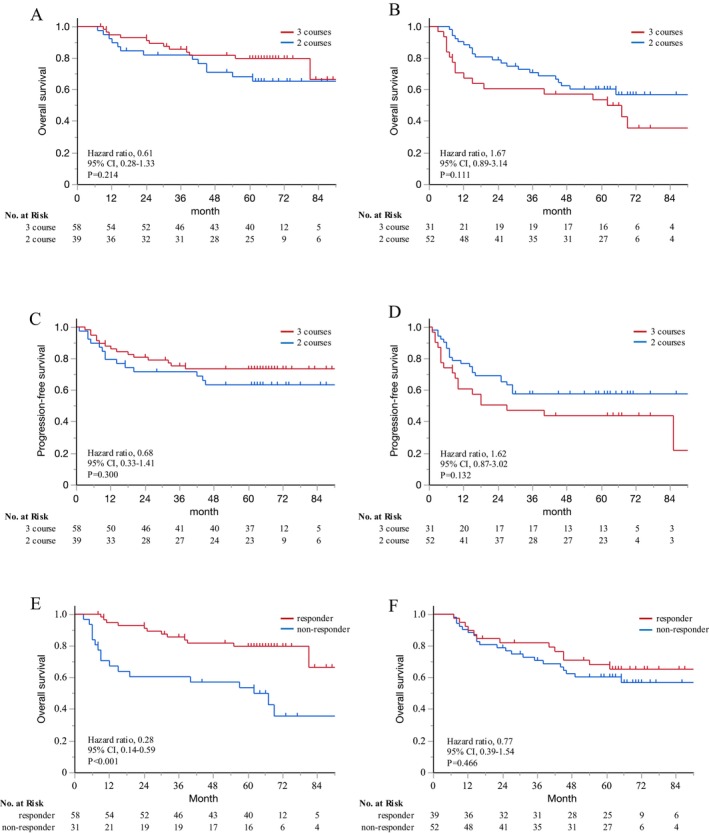
The results of survival analysis based on clinical treatment responses, categorizing patients into responders and non‐responders and comparing two versus three courses of DCF, are presented as follows: (A) overall survival among responders, (B) overall survival among non‐responders, (C) progression‐free survival among responders, and (D) progression‐free survival among non‐responders. A comparison between responders and non‐responders in each group is presented as follows: (E) overall survival in the three course DCF group and (F) overall survival in the two course DCF group.

Next, long‐term survival was compared based on clinical treatment responses, categorizing patients as responders or non‐responders. Among the responders, the 5‐year OS rates were 79.8% in the three course DCF group and 68.2% in the two course DCF group (HR: 0.61, 95% CI: 0.28–1.33, *p* = 0.214; Figure 4A). In contrast, among non‐responders, the 5‐year OS tended to be worse in the three course DCF group compared to the two course DCF group (5‐year OS: 53.7% vs. 60.4%, HR: 1.67, 95% CI: 0.89–3.14, *p* = 0.111; Figure 4B). PFS showed a similar trend to OS: the responders exhibited an HR of 0.68 (95% CI: 0.33–1.41, *p* = 0.300; Figure 4C), whereas the non‐responders showed an HR of 1.62 (95% CI: 0.87–3.02, *p* = 0.132; Figure 4D). Among patients receiving three courses of DCF, non‐responders had significantly worse 5‐year OS compared with responders (5‐year OS: 79.8% vs. 50.7%, HR: 0.28, 95% CI: 0.14–0.59, *p* < 0.001; Figure 4E). By contrast, no significant differences were found in long‐term survival between responders and non‐responders in the two course DCF group (5‐year OS: 68.2% vs. 60.4%, HR: 0.77, 95% CI: 0.39–1.54, *p* = 0.466; Figure 4F).

The potential factors for OS were investigated in each group. In the three course DCF group, multivariate analysis identified age, cT stage, clinical response, and pN stage as independent factors for OS (*p* < 0.001, *p* = 0.004, *p* < 0.001, and *p* = 0.029, respectively). In the two course DCF group, multivariate analysis revealed the completion of planned courses as an independent factor for OS (*p* = 0.010) (Table 3).

**TABLE 3:** Univariate and multivariate Cox model analysis for overall survival in patients.

		2‐couse DCF						3‐couse DCF					
		Univariate			Multivariate			Univariate			Multivariate		
		OR	95%CI	*p*	OR	95%CI	*p*	OR	95%CI	*p*	OR	95%CI	*p*
Age	< 65 y vs. 65 y ≦	1.47	[0.73–2.97]	0.285				0.22	[0.08–0.64]	0.005	0.08	[0.02–0.35]	< 0.001
Sex	female vs. male	0.54	[0.16–1.77]	0.308				1.45	[0.55–3.81]	0.454			
Performance status	0 vs. 1	0.91	[0.32–2.59]	0.860				1.17	[0.34–4.01]	0.809			
Pretreatment BMI	< 18.5 vs. 18.5 ≦	0.46	[0.19–1.25]	0.136				0.65	[0.25–1.72]	0.386			
Tumor marker SCC	< 1.6 vs. 1.6 ≦	0.88	[0.43–1.78]	0.716				0.8	[0.38–1.67]	0.554			
Tumor location	Upper vs. Middle/Lower	0.77	[0.30–1.99]	0.589				0.73	[0.25–2.09]	0.554			
cT stage	1–2 vs. 3–4	0.5	[0.22–1.15]	0.103				0.38	[0.15–0.99]	0.049	0.12	[0.03–0.51]	0.004
cN stage	Negative vs. positive	0.47	[0.16–1.33]	0.154				1.36	[0.60–3.05]	0.459			
cStage	I‐II vs. III‐IV	0.47	[0.21–1.05]	0.066	0.85	[0.29–2.49]	0.773	0.64	[0.29–1.41]	0.269			
Dose reduction	Full dose vs. reduce/stop	0.14	[0.02–1.06]	0.057	0.34	[0.04–2.67]	0.306	0.29	[0.04–2.16]	0.228			
Completion of planned courses	Completed vs. not completed	0.24	[0.11–0.50]	< 0.001	0.30	[0.12–0.76]	0.010	0.76	[0.31–1.88]	0.552			
Clinical treatment response	Responder vs. non‐responder	0.84	[0.43–1.64]	0.606				0.3	[0.14–0.61]	0.001	0.22	[0.09–0.51]	< 0.001
Surgical curability	R0 vs. non‐R0/unresectable	0.1	[0.04–0.28]	< 0.001	0.32	[0.63–5.12]	0.202	0.06	[0.02–0.16]	< 0.001	0.26	[0.04–1.67]	0.155
pT stage	1–2 vs. 3–4	0.22	[0.10–0.50]	< 0.001	0.56	[0.20–1.59]	0.274	0.47	[0.22–1.02]	0.056	0.85	[0.25–2.83]	0.790
pN stage	0–1 vs. 2–3	0.26	[0.13–0.54]	< 0.001	0.41	[0.15–1.14]	0.088	0.36	[0.16–0.81]	0.013	0.26	[0.08–0.87]	0.029
pStage	I‐II vs. III‐IV	0.2	[0.10–0.43]	< 0.001	0.60	[0.19–1.93]	0.393	0.41	[0.18–0.94]	0.034	0.88	[0.22–3.52]	0.854
Histopathological response	Grade 2–3 vs. 0–1	0.29	[0.12–0.70]	0.006	0.60	[0.23–1.55]	0.291	0.44	[0.18–1.05]	0.065	0.48	[0.17–1.32]	0.155

Abbreviations: BMI, body mass index; SCC, squamous cell carcinoma.

## 4 Discussion

Preoperative treatment followed by surgery has become a widely accepted strategy for treating locally advanced esophageal cancer. Preoperative chemotherapy with the DCF regimen is considered a standard neoadjuvant treatment for locally advanced ESCC [5, 11, 12]. However, the optimal number of NAC cycles for the DCF regimen has not yet been established. This multi‐institutional, randomized phase II trial is the first to demonstrate comparable 5‐year OS and PFS rates between two and three courses of DCF in both ITT and per‐protocol analyses. The pattern of postoperative recurrence was similar between the two groups. Subgroup analysis revealed that non‐responders to three courses of DCF had significantly worse long‐term survival outcomes, whereas this tendency was less pronounced in the two course group. Conversely, three courses of DCF may confer favorable long‐term survival outcomes in patients aged < 65 years or in those with clinical treatment responses. This randomized trial is the first to compare long‐term survival outcomes across different cycles of neoadjuvant DCF therapy for locally advanced esophageal cancer.

Previous studies have demonstrated the long‐term survival benefits of preoperative chemotherapy followed by radical esophagectomy. A large randomized clinical trial (JCOG9907) previously demonstrated that two courses of CF administered preoperatively significantly improved long‐term survival compared with two courses of CF administered postoperatively for locally advanced ESCC. This trial reported a 5‐year OS rate of 43% in patients with preoperative CF [10]. To further enhance the response rate to NAC and improve survival outcomes, the OGSG1003 trial compared two courses of triplet NAC regimens: DCF and CF combined with adriamycin (ACF) [12]. This trial found that DCF was associated with better long‐term survival compared with ACF in patients with resectable ESCC, with 5‐year OS rates of 63.5% for DCF and 49.4% for ACF [11]. Based on these findings, we hypothesized that an additional course of DCF could further improve survival outcomes by eradicating micrometastases beyond the surgical field. In this study, the 5‐year OS rates of the two course DCF were 63.8% in the ITT analysis and 68.8% in the per‐protocol analysis, which were comparable to the OGSG1003 trial results. Although the three course DCF exhibited slightly better long‐term survival outcomes compared with the two course DCF (5‐year OS: 70.7% vs. 63.8%), the additional course of DCF did not lead to a statistically significant improvement in long‐term survival (HR: 0.91, *p* = 0.717). Recently, a randomized, controlled, open‐label, phase 3 trial (JCOG1109) compared the efficacy and safety of two courses of CF, three courses of DCF, and two courses of CF combined with RT in locally advanced ESCC. This study demonstrated a statistically significant OS benefit for three courses of DCF followed by esophagectomy compared with two courses of CF. Although the study did not report the 5‐year survival rates, 3‐year OS rates were 72.1% for three courses of DCF and 62.6% for two courses of CF [5]. In this study, the 3‐year OS rates were 76.9% for three courses of DCF and 75.6% for two courses of DCF. Thus, the long‐term survival outcomes of the three courses of DCF in the present study appear comparable to those reported in the JCOG1109 trial.

Our previous study on short‐term outcomes found that two and three courses of DCF in the NAC setting were equally feasible. An additional course of DCF was associated with a better clinical response rate and a relatively higher pathological CR rate for the primary tumor [13]. By contrast, the pT stage, pN stage, lymphatic invasion, venous invasion, surgical outcomes, and overall pathological responses to NAC were similar between the two groups [14]. Miyata et al. investigated the impact of pathological tumor regression and the number of involved lymph nodes on survival and the occurrence of systemic disease in 405 patients with ESCC who received NAC. This study demonstrated that the post‐treatment nodal status is a useful predictor of prognosis and systemic disease occurrence [20]. Additionally, numerous studies have shown that patients with lymph node metastasis experience poorer survival outcomes [27, 28, 29]. Although three courses of DCF resulted in better clinical treatment responses compared with that of the two courses, the absence of differences in pathological results, including lymph node metastasis [14], between the two groups may explain the similar long‐term prognoses and recurrence patterns in both groups.

In the present study, a subgroup analysis showed that non‐responders to three courses of DCF tended to have worse long‐term survival compared with those who received two courses of DCF. Furthermore, among patients who received three courses of DCF, non‐responders exhibited significantly worse long‐term survival compared with responders (HR: 0.28, *p* < 0.001). Multivariate analysis of the three course DCF group demonstrated that clinical response was an independent factor for OS. However, this trend was less pronounced in the two course group. These findings suggest that administering an additional course of DCF chemotherapy to non‐responders may negatively affect long‐term survival. Our previous exploratory analysis examined the association between tumor response and survival outcomes in patients receiving three courses of DCF chemotherapy. Approximately 40% of the patients exhibited less than 10% tumor reduction during the third course, which was independently associated with poorer survival outcomes compared with those with more than 10% tumor reduction. The reduction rate of the primary tumor after the first two courses (tumor reduction rate < 50%) was most strongly correlated with poor tumor response during the third course (reduction rate < 10%) [30]. These results suggest that continuation of NAC until the three courses may worsen the survival of patients who experienced a tumor reduction rate of < 50% after the first two courses.

In contrast to the general findings, certain subgroups showed long‐term survival benefits after receiving three courses of DCF chemotherapy. Specifically, patients aged ≤ 65 years who received three courses of DCF had significantly better long‐term survival compared with those who received two courses. These results are consistent with the findings of our previous study [14]. Multivariate analysis of OS in the three course DCF group further supported these results. These findings suggest that patients aged < 65 years may be suitable candidates for receiving three courses of DCF. Based on these results and our previous study [13, 14, 30], two courses of preoperative DCF followed by radical esophagectomy may be one of the treatment approaches for patients with locally advanced ESCC. Furthermore, DCF therapy as neoadjuvant therapy has the potential to be individualized and optimized by age and accurate assessment of imaging after the first two courses of DCF.

This study was a phase II trial with a small sample size, which limited the ability to establish definitive standards for preoperative treatment. Recently, the JCOG1109 trial provided robust evidence supporting favoring three courses of DCF over two courses of CF. Exploratory analyses of the JCOG1109 data are expected to help refine the optimal number of courses of neoadjuvant chemotherapy.

In conclusion, this study is the first to demonstrate that two courses of preoperative DCF achieve long‐term survival outcomes comparable to those of three courses. For patients with locally advanced ESCC, two courses of preoperative DCF followed by radical esophagectomy can be one of the potential treatment strategies.

## Author Contributions

**Takahito Sugase:** data curation, writing – original draft. **Hiroshi Miyata:** data curation, writing – review and editing. **Takashi Kanemura:** data curation. **Norihiro Matsuura:** data curation. **Tomoki Makino:** data curation, project administration, resources. **Makoto Yamasaki:** data curation. **Koji Tanaka:** data curation. **Kotaro Yamashita:** data curation. **Kota Momose:** data curation. **Osamu Shiraishi:** data curation, project administration. **Keijiro Sugimura:** data curation. **Masaaki Motoori:** data curation. **Kazumasa Fujitani:** data curation. **Atsushi Takeno:** data curation. **Motohiro Hirao:** data curation. **Yutaka Kimura:** data curation. **Taroh Satoh:** data curation. **Masahiko Yano:** data curation. **Yuichiro Doki:** conceptualization, project administration. **Takushi Yasuda:** conceptualization, project administration.

## Ethics Statement

The study was approved by the institutional review boards of the six participating hospitals prior to patient enrollment. The study was conducted in accordance with the principles of the Helsinki Declaration and registered with the University Hospital Medical Information Network Clinical Trials Registry.

## Conflicts of Interest

Yuichiro Doki is an Editorial Board member of the Annals of Gastroenterological Surgery. All remaining authors declare no conflict of interests for this article.
